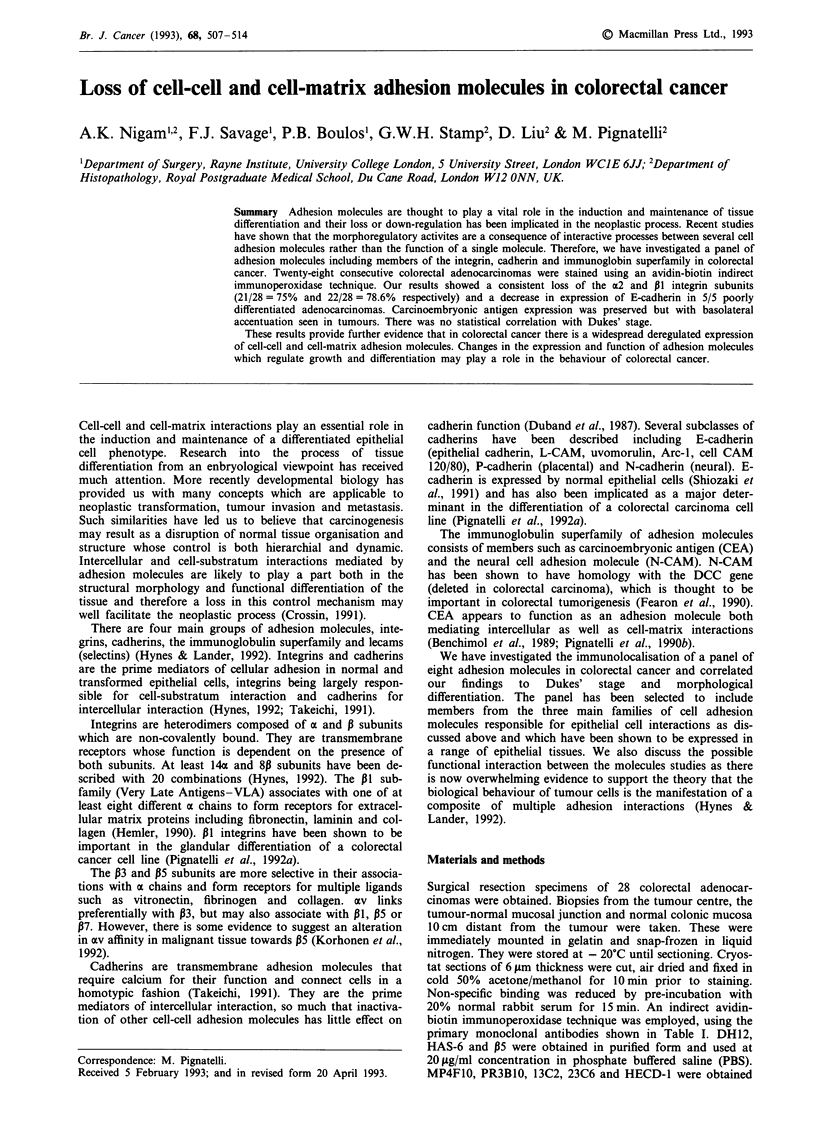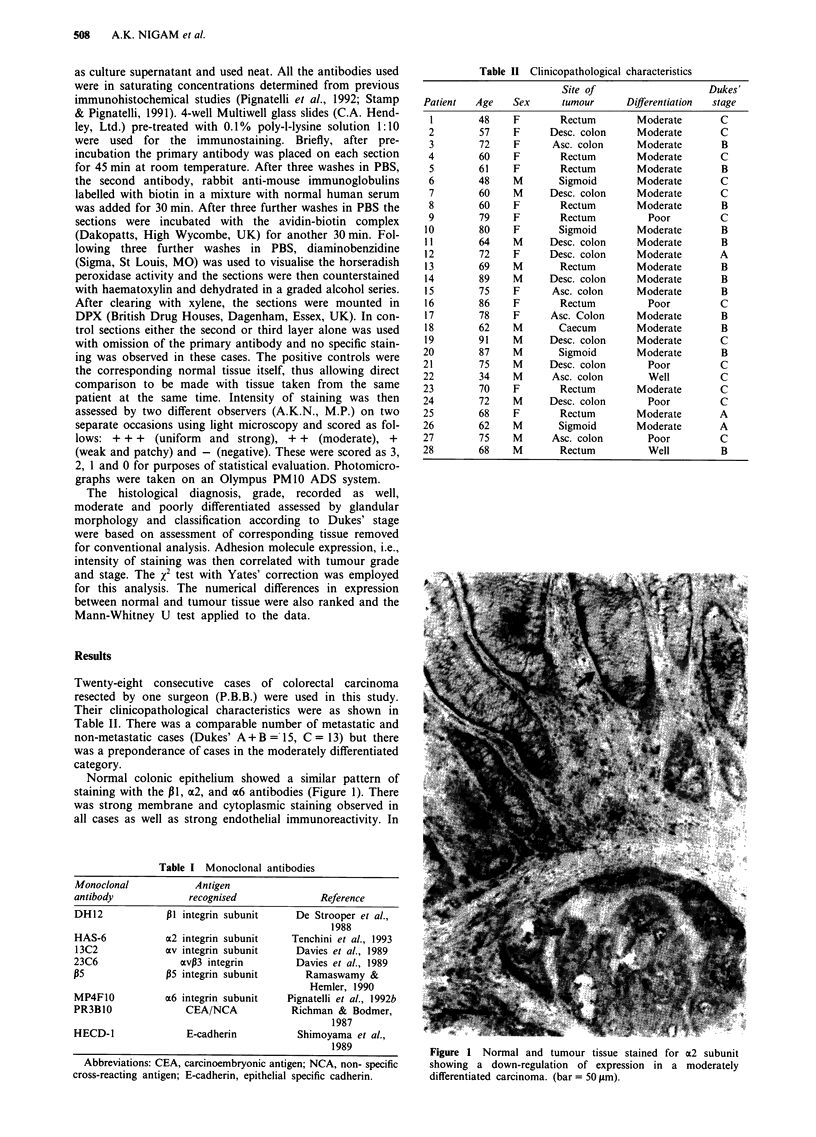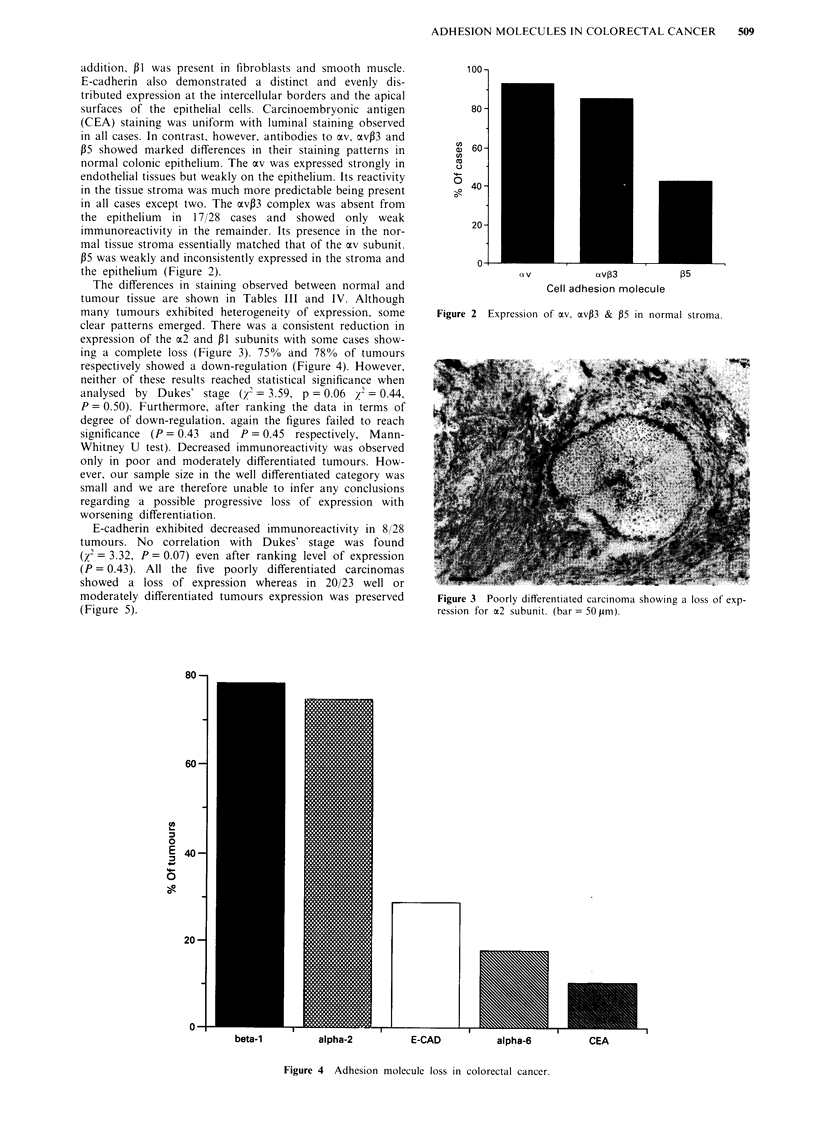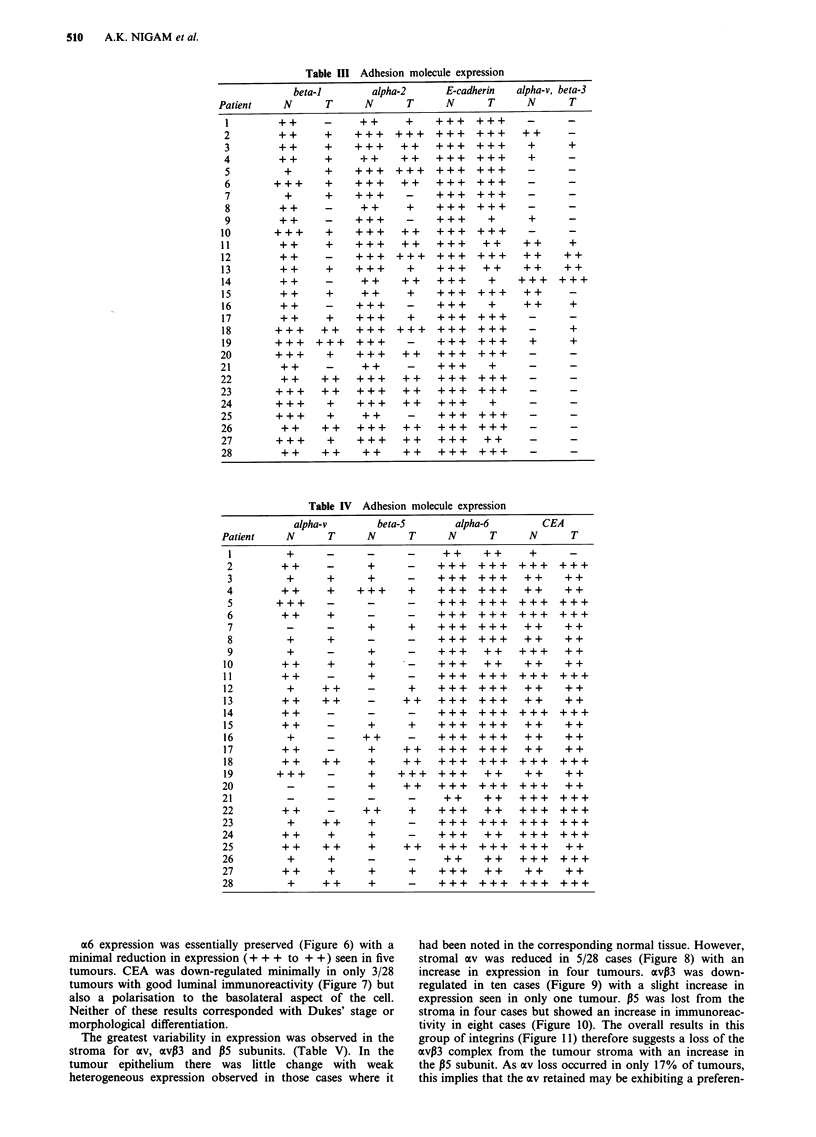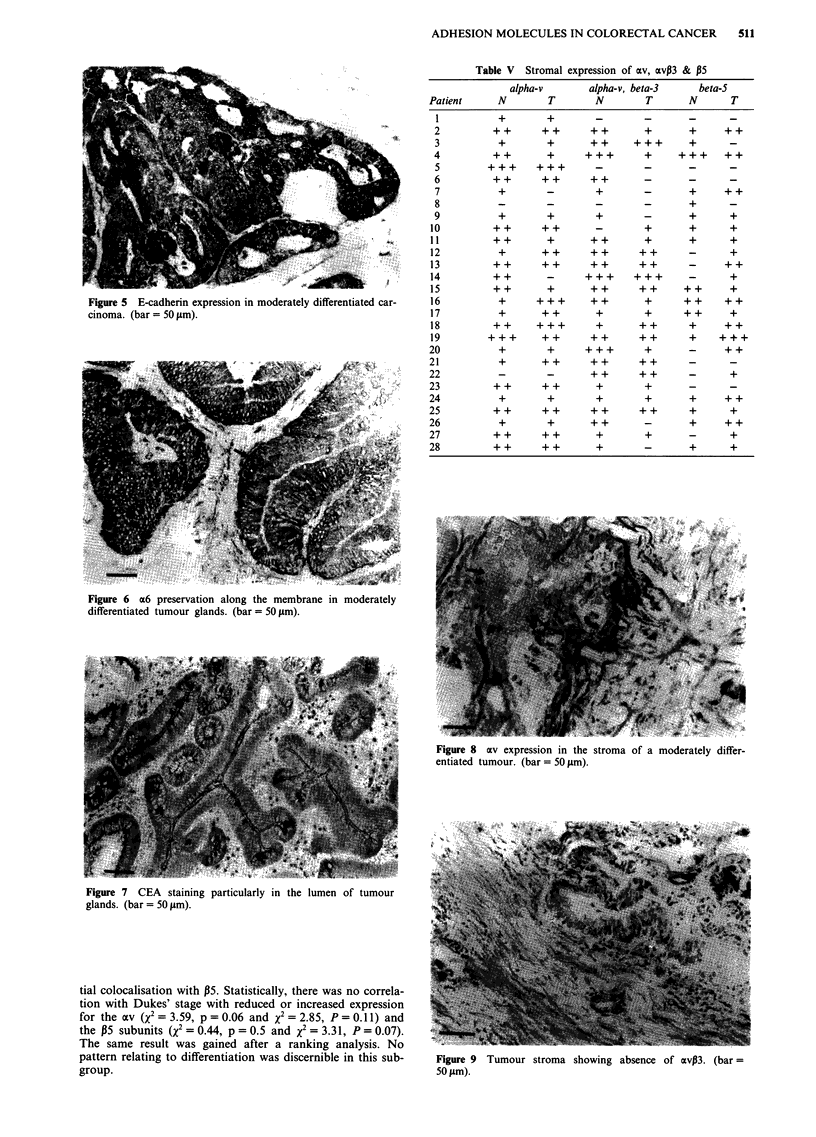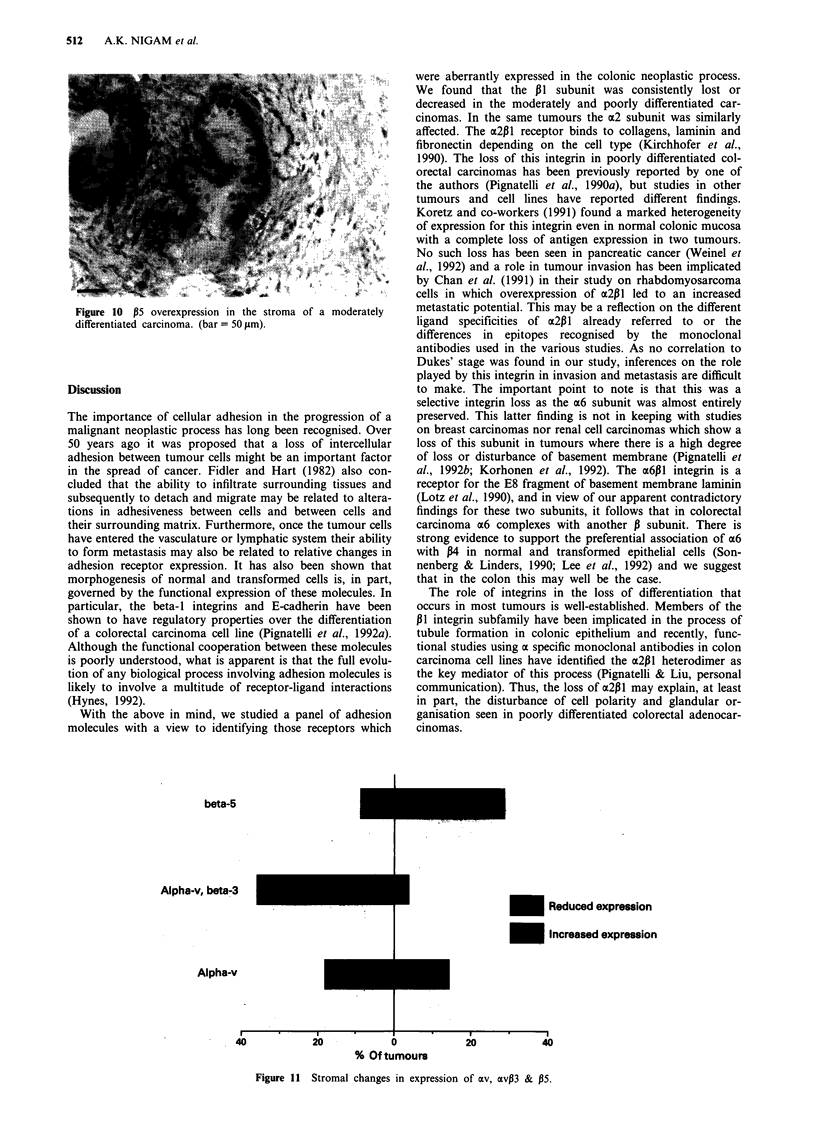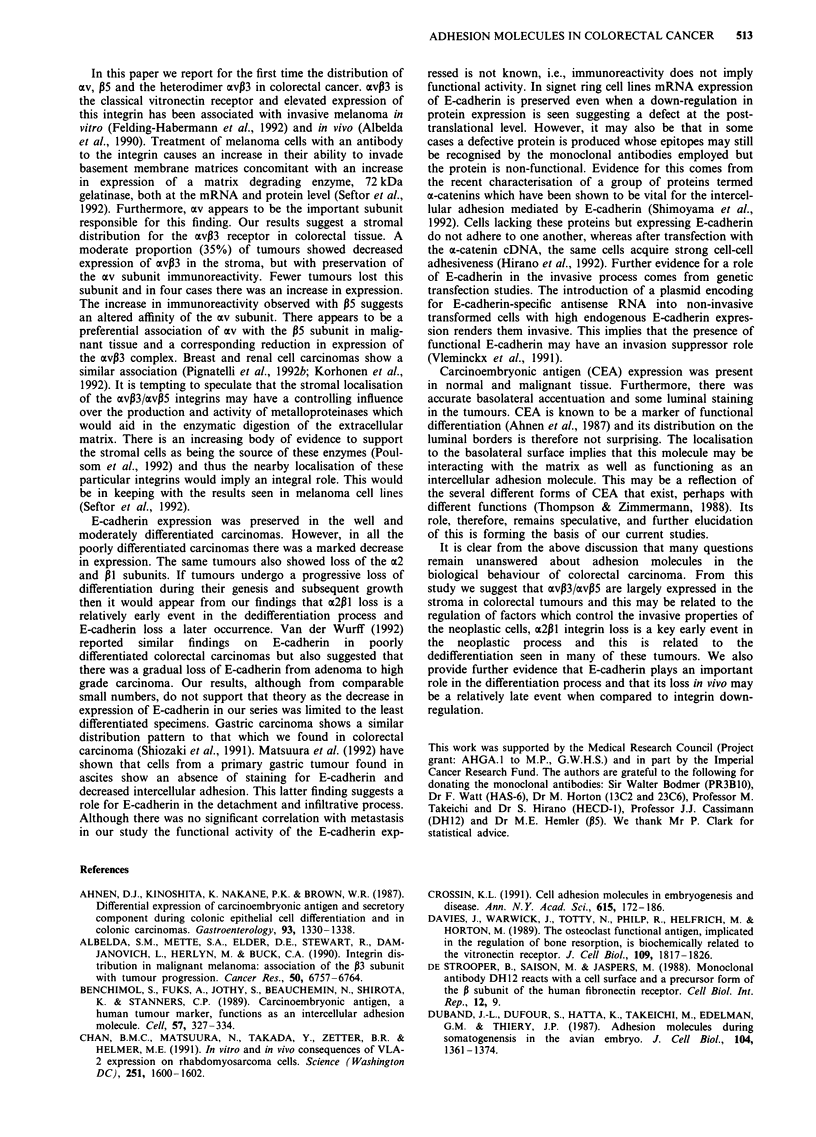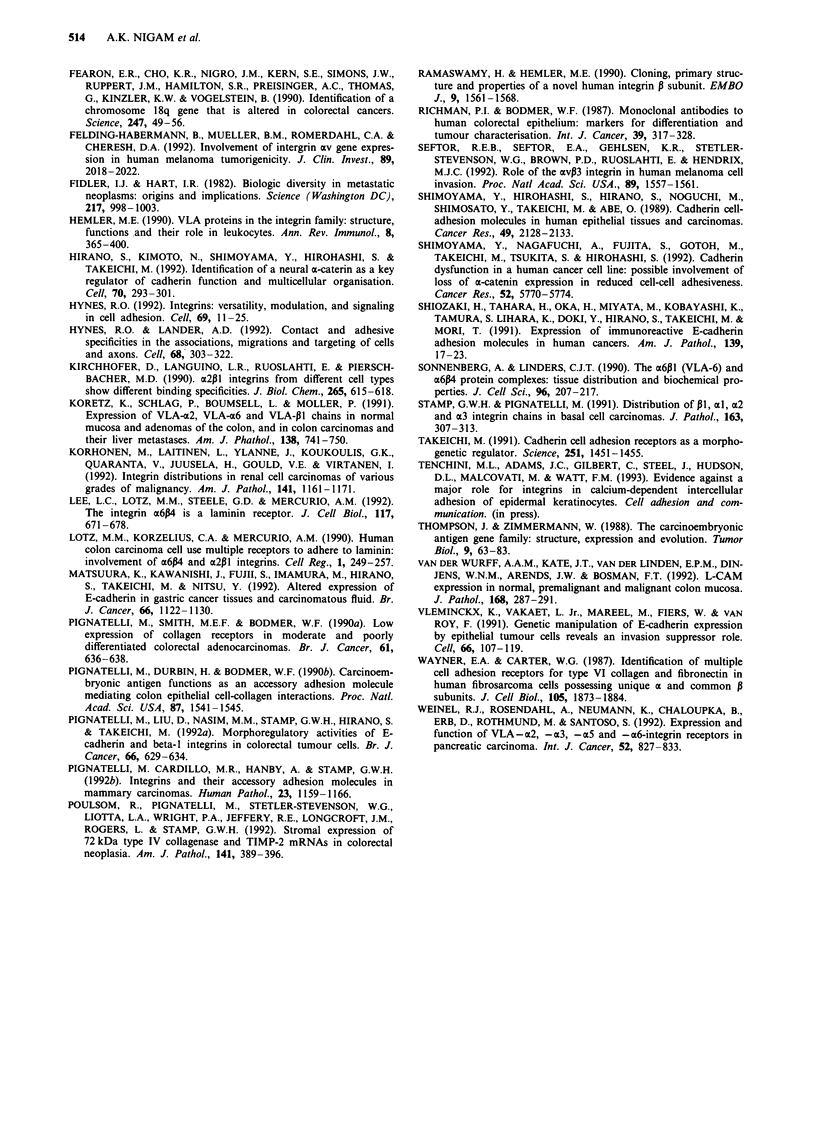# Loss of cell-cell and cell-matrix adhesion molecules in colorectal cancer.

**DOI:** 10.1038/bjc.1993.377

**Published:** 1993-09

**Authors:** A. K. Nigam, F. J. Savage, P. B. Boulos, G. W. Stamp, D. Liu, M. Pignatelli

**Affiliations:** Department of Surgery, Rayne Institute, University College London, UK.

## Abstract

**Images:**


*Br. J. Cancer* (1993), **68**, 507–514 © Macmillan Press Ltd., 1993

### Loss of cell-cell and cell-matrix adhesion molecules in colorectal cancer

A.K. Nigam[1,2], F.J. Savage[1], P.B. Boulos[1], G.W.H. Stamp[2], D. Liu[2] & M. Pignatelli[2]

[1]*Department of Surgery, Rayne Institute, University College London, 5 University Street, London WC1E 6JJ;* [2]*Department of Histopathology, Royal Postgraduate Medical School, Du Cane Road, London W12 0NN, UK.*

**Summary** Adhesion molecules are thought to play a vital role in the induction and maintenance of tissue differentiation and their loss or down-regulation has been implicated in the neoplastic process. Recent studies have shown that the morphoregulatory activites are a consequence of interactive processes between several cell adhesion molecules rather than the function of a single molecule. Therefore, we have investigated a panel of adhesion molecules including members of the integrin, cadherin and immunoglobin superfamily in colorectal cancer. Twenty-eight consecutive colorectal adenocarcinomas were stained using an avidin-biotin indirect immunoperoxidase technique. Our results showed a consistent loss of the α2 and β1 integrin subunits (21/28 = 75% and 22/28 = 78.6% respectively) and a decrease in expression of E-cadherin in 5/5 poorly differentiated adenocarcinomas. Carcinoembryonic antigen expression was preserved but with basolateral accentuation seen in tumours. There was no statistical correlation with Dukes' stage.

These results provide further evidence that in colorectal cancer there is a widespread deregulated expression of cell-cell and cell-matrix adhesion molecules. Changes in the expression and function of adhesion molecules which regulate growth and differentiation may play a role in the behaviour of colorectal cancer.

Cell-cell and cell-matrix interactions play an essential role in the induction and maintenance of a differentiated epithelial cell phenotype. Research into the process of tissue differentiation from an enbryological viewpoint has received much attention. More recently developmental biology has provided us with many concepts which are applicable to neoplastic transformation, tumour invasion and metastasis. Such similarities have led us to believe that carcinogenesis may result as a disruption of normal tissue organisation and structure whose control is both hierarchial and dynamic. Intercellular and cell-substratum interactions mediated by adhesion molecules are likely to play a part both in the structural morphology and functional differentiation of the tissue and therefore a loss in this control mechanism may well facilitate the neoplastic process (Crossin, 1991).

There are four main groups of adhesion molecules, integrins, cadherins, the immunoglobulin superfamily and lecams (selectins) (Hynes & Lander, 1992). Integrins and cadherins are the prime mediators of cellular adhesion in normal and transformed epithelial cells, integrins being largely responsible for cell-substratum interaction and cadherins for intercellular interaction (Hynes, 1992; Takeichi, 1991).

Integrins are heterodimers composed of α and β subunits which are non-covalently bound. They are transmembrane receptors whose function is dependent on the presence of both subunits. At least 14α and 8β subunits have been described with 20 combinations (Hynes, 1992). The β1 subfamily (Very Late Antigens–VLA) associates with one of at least eight different α chains to form receptors for extracellular matrix proteins including fibronectin, laminin and collagen (Hemler, 1990). β1 integrins have been shown to be important in the glandular differentiation of a colorectal cancer cell line (Pignatelli *et al.*, 1992*a*).

The β3 and β5 subunits are more selective in their associations with α chains and form receptors for multiple ligands such as vitronectin, fibrinogen and collagen. αv links preferentially with β3, but may also associate with β1, β5 or β7. However, there is some evidence to suggest an alteration in αv affinity in malignant tissue towards β5 (Korhonen *et al.*, 1992).

Cadherins are transmembrane adhesion molecules that require calcium for their function and connect cells in a homotypic fashion (Takeichi, 1991). They are the prime mediators of intercellular interaction, so much that inactivation of other cell-cell adhesion molecules has little effect on cadherin function (Duband *et al.*, 1987). Several subclasses of cadherins have been described including E-cadherin (epithelial cadherin, L-CAM, uvomorulin, Arc-1, cell CAM 120/80), P-cadherin (placental) and N-cadherin (neural). E-cadherin is expressed by normal epithelial cells (Shiozaki *et al.*, 1991) and has also been implicated as a major determinant in the differentiation of a colorectal carcinoma cell line (Pignatelli *et al.*, 1992*a*).

The immunoglobulin superfamily of adhesion molecules consists of members such as carcinoembryonic antigen (CEA) and the neural cell adhesion molecule (N-CAM). N-CAM has been shown to have homology with the DCC gene (deleted in colorectal carcinoma), which is thought to be important in colorectal tumorigenesis (Fearon *et al.*, 1990). CEA appears to function as an adhesion molecule both mediating intercellular as well as cell-matrix interactions (Benchimol *et al.*, 1989; Pignatelli *et al.*, 1990*b*).

We have investigated the immunolocalisation of a panel of eight adhesion molecules in colorectal cancer and correlated our findings to Dukes' stage and morphological differentiation. The panel has been selected to include members from the three main families of cell adhesion molecules responsible for epithelial cell interactions as discussed above and which have been shown to be expressed in a range of epithelial tissues. We also discuss the possible functional interaction between the molecules studies as there is now overwhelming evidence to support the theory that the biological behaviour of tumour cells is the manifestation of a composite of multiple adhesion interactions (Hynes & Lander, 1992).

### Materials and methods

Surgical resection specimens of 28 colorectal adenocarcinomas were obtained. Biopsies from the tumour centre, the tumour-normal mucosal junction and normal colonic mucosa 10 cm distant from the tumour were taken. These were immediately mounted in gelatin and snap-frozen in liquid nitrogen. They were stored at − 20°C until sectioning. Cryostat sections of 6 μm thickness were cut, air dried and fixed in cold 50% acetone/methanol for 10 min prior to staining. Non-specific binding was reduced by pre-incubation with 20% normal rabbit serum for 15 min. An indirect avidin-biotin immunoperoxidase technique was employed, using the primary monoclonal antibodies shown in Table I. DH12, HAS-6 and β5 were obtained in purified form and used at 20 μg/ml concentration in phosphate buffered saline (PBS). MP4F10, PR3B10, 13C2, 23C6 and HECD-1 were obtained

Correspondence: M. Pignatelli.
Received 5 February 1993; and in revised form 20 April 1993.

as culture supernatant and used neat. All the antibodies used were in saturating concentrations determined from previous immunohistochemical studies (Pignatelli *et al.*, 1992; Stamp & Pignatelli, 1991). 4-well Multiwell glass slides (C.A. Hendley, Ltd.) pre-treated with 0.1% poly-l-lysine solution 1:10 were used for the immunostaining. Briefly, after pre-incubation the primary antibody was placed on each section for 45 min at room temperature. After three washes in PBS, the second antibody, rabbit anti-mouse immunoglobulins labelled with biotin in a mixture with normal human serum was added for 30 min. After three further washes in PBS the sections were incubated with the avidin-biotin complex (Dakopatts, High Wycombe, UK) for another 30 min. Following three further washes in PBS, diaminobenzidine (Sigma, St Louis, MO) was used to visualise the horseradish peroxidase activity and the sections were then counterstained with haematoxylin and dehydrated in a graded alcohol series. After clearing with xylene, the sections were mounted in DPX (British Drug Houses, Dagenham, Essex, UK). In control sections either the second or third layer alone was used with omission of the primary antibody and no specific staining was observed in these cases. The positive controls were the corresponding normal tissue itself, thus allowing direct comparison to be made with tissue taken from the same patient at the same time. Intensity of staining was then assessed by two different observers (A.K.N., M.P.) on two separate occasions using light microscopy and scored as follows: + + + (uniform and strong), + + (moderate), + (weak and patchy) and − (negative). These were scored as 3, 2, 1 and 0 for purposes of statistical evaluation. Photomicrographs were taken on an Olympus PM10 ADS system.

The histological diagnosis, grade, recorded as well, moderate and poorly differentiated assessed by glandular morphology and classification according to Dukes' stage were based on assessment of corresponding tissue removed for conventional analysis. Adhesion molecule expression, i.e., intensity of staining was then correlated with tumour grade and stage. The $\chi^2$ test with Yates' correction was employed for this analysis. The numerical differences in expression between normal and tumour tissue were also ranked and the Mann-Whitney U test applied to the data.

### Results

Twenty-eight consecutive cases of colorectal carcinoma resected by one surgeon (P.B.B.) were used in this study. Their clinicopathological characteristics were as shown in Table II. There was a comparable number of metastatic and non-metastatic cases (Dukes' A + B = 15, C = 13) but there was a preponderance of cases in the moderately differentiated category.

Normal colonic epithelium showed a similar pattern of staining with the β1, α2, and α6 antibodies (Figure 1). There was strong membrane and cytoplasmic staining observed in all cases as well as strong endothelial immunoreactivity. In

**Table I** Monoclonal antibodies

| *Monoclonal antibody* | *Antigen recognised* | *Reference* |
|---|---|---|
| DH12 | β1 integrin subunit | De Strooper *et al.*, 1988 |
| HAS-6 | α2 integrin subunit | Tenchini *et al.*, 1993 |
| 13C2 | αv integrin subunit | Davies *et al.*, 1989 |
| 23C6 | αvβ3 integrin | Davies *et al.*, 1989 |
| β5 | β5 integrin subunit | Ramaswamy & Hemler, 1990 |
| MP4F10 | α6 integrin subunit | Pignatelli *et al.*, 1992*b* |
| PR3B10 | CEA/NCA | Richman & Bodmer, 1987 |
| HECD-1 | E-cadherin | Shimoyama *et al.*, 1989 |

Abbreviations: CEA, carcinoembryonic antigen; NCA, non- specific cross-reacting antigen; E-cadherin, epithelial specific cadherin.

**Table II** Clinicopathological characteristics

| *Patient* | *Age* | *Sex* | *Site of tumour* | *Differentiation* | *Dukes' stage* |
|---|---|---|---|---|---|
| 1 | 48 | F | Rectum | Moderate | C |
| 2 | 57 | F | Desc. colon | Moderate | C |
| 3 | 72 | F | Asc. colon | Moderate | B |
| 4 | 60 | F | Rectum | Moderate | C |
| 5 | 61 | F | Rectum | Moderate | B |
| 6 | 48 | M | Sigmoid | Moderate | C |
| 7 | 60 | M | Desc. colon | Moderate | C |
| 8 | 60 | F | Rectum | Moderate | B |
| 9 | 79 | F | Rectum | Poor | C |
| 10 | 80 | F | Sigmoid | Moderate | B |
| 11 | 64 | M | Desc. colon | Moderate | B |
| 12 | 72 | F | Desc. colon | Moderate | A |
| 13 | 69 | M | Rectum | Moderate | B |
| 14 | 89 | M | Desc. colon | Moderate | B |
| 15 | 75 | F | Asc. colon | Moderate | B |
| 16 | 86 | F | Rectum | Poor | C |
| 17 | 78 | F | Asc. Colon | Moderate | B |
| 18 | 62 | M | Caecum | Moderate | B |
| 19 | 91 | M | Desc. colon | Moderate | C |
| 20 | 87 | M | Sigmoid | Moderate | B |
| 21 | 75 | M | Desc. colon | Poor | C |
| 22 | 34 | M | Asc. colon | Well | C |
| 23 | 70 | F | Rectum | Moderate | C |
| 24 | 72 | M | Desc. colon | Poor | C |
| 25 | 68 | F | Rectum | Moderate | A |
| 26 | 62 | M | Sigmoid | Moderate | A |
| 27 | 75 | M | Asc. colon | Poor | C |
| 28 | 68 | M | Rectum | Well | B |

**Figure 1** Normal and tumour tissue stained for α2 subunit showing a down-regulation of expression in a moderately differentiated carcinoma. (bar = 50 μm).

addition, $\beta1$ was present in fibroblasts and smooth muscle. E-cadherin also demonstrated a distinct and evenly distributed expression at the intercellular borders and the apical surfaces of the epithelial cells. Carcinoembryonic antigen (CEA) staining was uniform with luminal staining observed in all cases. In contrast, however, antibodies to $\alpha v$, $\alpha v\beta3$ and $\beta5$ showed marked differences in their staining patterns in normal colonic epithelium. The $\alpha v$ was expressed strongly in endothelial tissues but weakly on the epithelium. Its reactivity in the tissue stroma was much more predictable being present in all cases except two. The $\alpha v\beta3$ complex was absent from the epithelium in 17/28 cases and showed only weak immunoreactivity in the remainder. Its presence in the normal tissue stroma essentially matched that of the $\alpha v$ subunit. $\beta5$ was weakly and inconsistently expressed in the stroma and the epithelium (Figure 2).

The differences in staining observed between normal and tumour tissue are shown in Tables III and IV. Although many tumours exhibited heterogeneity of expression, some clear patterns emerged. There was a consistent reduction in expression of the $\alpha2$ and $\beta1$ subunits with some cases showing a complete loss (Figure 3). 75% and 78% of tumours respectively showed a down-regulation (Figure 4). However, neither of these results reached statistical significance when analysed by Dukes' stage ($\chi^2 = 3.59$, p = 0.06 $\chi^2 = 0.44$, $P = 0.50$). Furthermore, after ranking the data in terms of degree of down-regulation, again the figures failed to reach significance ($P = 0.43$ and $P = 0.45$ respectively, Mann-Whitney U test). Decreased immunoreactivity was observed only in poor and moderately differentiated tumours. However, our sample size in the well differentiated category was small and we are therefore unable to infer any conclusions regarding a possible progressive loss of expression with worsening differentiation.

E-cadherin exhibited decreased immunoreactivity in 8/28 tumours. No correlation with Dukes' stage was found ($\chi^2 = 3.32$, $P = 0.07$) even after ranking level of expression ($P = 0.43$). All the five poorly differentiated carcinomas showed a loss of expression whereas in 20/23 well or moderately differentiated tumours expression was preserved (Figure 5).

**Figure 2** Expression of $\alpha v$, $\alpha v\beta3$ & $\beta5$ in normal stroma.

**Figure 3** Poorly differentiated carcinoma showing a loss of expression for $\alpha2$ subunit. (bar = 50 $\mu$m).

**Figure 4** Adhesion molecule loss in colorectal cancer.

**Table III** Adhesion molecule expression

| | *beta-1* | | *alpha-2* | | *E-cadherin* | | *alpha-v, beta-3* | |
|---|---|---|---|---|---|---|---|---|
| *Patient* | *N* | *T* | *N* | *T* | *N* | *T* | *N* | *T* |
| 1 | ++ | – | ++ | + | +++ | +++ | – | – |
| 2 | ++ | + | +++ | +++ | +++ | +++ | ++ | – |
| 3 | ++ | + | +++ | ++ | +++ | +++ | + | + |
| 4 | ++ | + | ++ | ++ | +++ | +++ | + | – |
| 5 | + | + | +++ | +++ | +++ | +++ | – | – |
| 6 | +++ | + | +++ | ++ | +++ | +++ | – | – |
| 7 | + | + | +++ | – | +++ | +++ | – | – |
| 8 | ++ | – | ++ | + | +++ | +++ | – | – |
| 9 | ++ | – | +++ | – | +++ | + | + | – |
| 10 | +++ | + | +++ | ++ | +++ | +++ | – | – |
| 11 | ++ | + | +++ | ++ | +++ | ++ | ++ | + |
| 12 | ++ | – | +++ | +++ | +++ | +++ | ++ | ++ |
| 13 | ++ | + | +++ | + | +++ | ++ | ++ | ++ |
| 14 | ++ | – | ++ | ++ | +++ | + | +++ | +++ |
| 15 | ++ | + | ++ | + | +++ | +++ | ++ | – |
| 16 | ++ | – | +++ | – | +++ | + | ++ | + |
| 17 | ++ | + | +++ | + | +++ | +++ | – | – |
| 18 | +++ | ++ | +++ | +++ | +++ | +++ | – | + |
| 19 | +++ | +++ | +++ | – | +++ | +++ | + | + |
| 20 | +++ | + | +++ | ++ | +++ | +++ | – | – |
| 21 | ++ | – | ++ | – | +++ | + | – | – |
| 22 | ++ | ++ | +++ | ++ | +++ | +++ | – | – |
| 23 | +++ | ++ | +++ | ++ | +++ | +++ | – | – |
| 24 | +++ | + | +++ | ++ | +++ | + | – | – |
| 25 | +++ | + | ++ | – | +++ | +++ | – | – |
| 26 | ++ | ++ | +++ | ++ | +++ | +++ | – | – |
| 27 | +++ | + | +++ | ++ | +++ | ++ | – | – |
| 28 | ++ | ++ | ++ | ++ | +++ | +++ | – | – |

**Table IV** Adhesion molecule expression

| | *alpha-v* | | *beta-5* | | *alpha-6* | | *CEA* | |
|---|---|---|---|---|---|---|---|---|
| *Patient* | *N* | *T* | *N* | *T* | *N* | *T* | *N* | *T* |
| 1 | + | – | – | – | ++ | ++ | + | – |
| 2 | ++ | – | + | – | +++ | +++ | +++ | +++ |
| 3 | + | + | + | – | +++ | +++ | ++ | ++ |
| 4 | ++ | + | +++ | + | +++ | +++ | ++ | ++ |
| 5 | +++ | – | – | – | +++ | +++ | +++ | +++ |
| 6 | ++ | + | – | – | +++ | +++ | +++ | +++ |
| 7 | – | – | + | + | +++ | +++ | ++ | ++ |
| 8 | + | + | – | – | +++ | +++ | ++ | ++ |
| 9 | + | – | + | – | +++ | ++ | +++ | ++ |
| 10 | ++ | + | + | – | +++ | ++ | ++ | ++ |
| 11 | ++ | – | + | – | +++ | +++ | +++ | +++ |
| 12 | + | ++ | – | + | +++ | +++ | ++ | ++ |
| 13 | ++ | ++ | – | ++ | +++ | +++ | ++ | ++ |
| 14 | ++ | – | – | – | +++ | +++ | +++ | +++ |
| 15 | ++ | – | + | + | +++ | +++ | ++ | ++ |
| 16 | + | – | ++ | – | +++ | +++ | ++ | ++ |
| 17 | ++ | – | + | ++ | +++ | +++ | ++ | ++ |
| 18 | ++ | ++ | + | ++ | +++ | +++ | +++ | +++ |
| 19 | +++ | – | + | +++ | +++ | ++ | ++ | ++ |
| 20 | – | – | + | ++ | +++ | +++ | +++ | ++ |
| 21 | – | – | – | – | ++ | ++ | +++ | +++ |
| 22 | ++ | – | ++ | + | +++ | ++ | +++ | +++ |
| 23 | + | ++ | + | – | +++ | +++ | +++ | +++ |
| 24 | ++ | + | + | – | +++ | ++ | +++ | +++ |
| 25 | ++ | ++ | + | ++ | +++ | +++ | +++ | ++ |
| 26 | + | + | – | – | ++ | ++ | +++ | +++ |
| 27 | ++ | + | + | + | +++ | ++ | ++ | ++ |
| 28 | + | ++ | + | – | +++ | +++ | +++ | +++ |

α6 expression was essentially preserved (Figure 6) with a minimal reduction in expression (+ + + to + +) seen in five tumours. CEA was down-regulated minimally in only 3/28 tumours with good luminal immunoreactivity (Figure 7) but also a polarisation to the basolateral aspect of the cell. Neither of these results corresponded with Dukes' stage or morphological differentiation.

The greatest variability in expression was observed in the stroma for αv, αvβ3 and β5 subunits. (Table V). In the tumour epithelium there was little change with weak heterogeneous expression observed in those cases where it had been noted in the corresponding normal tissue. However, stromal αv was reduced in 5/28 cases (Figure 8) with an increase in expression in four tumours. αvβ3 was down-regulated in ten cases (Figure 9) with a slight increase in expression seen in only one tumour. β5 was lost from the stroma in four cases but showed an increase in immunoreactivity in eight cases (Figure 10). The overall results in this group of integrins (Figure 11) therefore suggests a loss of the αvβ3 complex from the tumour stroma with an increase in the β5 subunit. As αv loss occurred in only 17% of tumours, this implies that the αv retained may be exhibiting a preferen-

**Figure 5** E-cadherin expression in moderately differentiated carcinoma. (bar = 50 μm).

**Figure 6** α6 preservation along the membrane in moderately differentiated tumour glands. (bar = 50 μm).

**Figure 7** CEA staining particularly in the lumen of tumour glands. (bar = 50 μm).

tial colocalisation with β5. Statistically, there was no correlation with Dukes' stage with reduced or increased expression for the αv ($\chi^2 = 3.59$, $p = 0.06$ and $\chi^2 = 2.85$, $P = 0.11$) and the β5 subunits ($\chi^2 = 0.44$, $p = 0.5$ and $\chi^2 = 3.31$, $P = 0.07$). The same result was gained after a ranking analysis. No pattern relating to differentiation was discernible in this subgroup.

**Table V** Stromal expression of αv, αvβ3 & β5

| | *alpha-v* | | *alpha-v, beta-3* | | *beta-5* | |
|---|---|---|---|---|---|---|
| *Patient* | *N* | *T* | *N* | *T* | *N* | *T* |
| 1 | + | + | − | − | − | − |
| 2 | ++ | ++ | ++ | + | + | ++ |
| 3 | + | + | ++ | +++ | + | − |
| 4 | ++ | + | +++ | + | +++ | ++ |
| 5 | +++ | +++ | − | − | − | − |
| 6 | ++ | ++ | ++ | − | − | − |
| 7 | + | − | + | − | + | ++ |
| 8 | − | − | − | − | + | − |
| 9 | + | + | + | − | + | + |
| 10 | ++ | ++ | − | + | + | + |
| 11 | ++ | + | ++ | + | + | + |
| 12 | + | ++ | ++ | ++ | − | + |
| 13 | ++ | ++ | ++ | ++ | − | ++ |
| 14 | ++ | − | +++ | +++ | − | + |
| 15 | ++ | + | ++ | ++ | ++ | + |
| 16 | + | +++ | ++ | + | ++ | ++ |
| 17 | + | ++ | + | + | ++ | + |
| 18 | ++ | +++ | + | ++ | + | ++ |
| 19 | +++ | ++ | ++ | ++ | + | +++ |
| 20 | + | + | +++ | + | − | ++ |
| 21 | + | ++ | ++ | ++ | − | − |
| 22 | − | − | ++ | ++ | − | + |
| 23 | ++ | ++ | + | + | − | − |
| 24 | + | + | + | + | + | ++ |
| 25 | ++ | ++ | ++ | ++ | + | + |
| 26 | + | + | ++ | − | + | ++ |
| 27 | ++ | ++ | + | + | − | + |
| 28 | ++ | ++ | + | − | + | + |

**Figure 8** αv expression in the stroma of a moderately differentiated tumour. (bar = 50 μm).

**Figure 9** Tumour stroma showing absence of αvβ3. (bar = 50 μm).

**Figure 10** $\beta 5$ overexpression in the stroma of a moderately differentiated carcinoma. (bar = 50 $\mu$m).

### Discussion

The importance of cellular adhesion in the progression of a malignant neoplastic process has long been recognised. Over 50 years ago it was proposed that a loss of intercellular adhesion between tumour cells might be an important factor in the spread of cancer. Fidler and Hart (1982) also concluded that the ability to infiltrate surrounding tissues and subsequently to detach and migrate may be related to alterations in adhesiveness between cells and between cells and their surrounding matrix. Furthermore, once the tumour cells have entered the vasculature or lymphatic system their ability to form metastasis may also be related to relative changes in adhesion receptor expression. It has also been shown that morphogenesis of normal and transformed cells is, in part, governed by the functional expression of these molecules. In particular, the beta-1 integrins and E-cadherin have been shown to have regulatory properties over the differentiation of a colorectal carcinoma cell line (Pignatelli *et al.*, 1992*a*). Although the functional cooperation between these molecules is poorly understood, what is apparent is that the full evolution of any biological process involving adhesion molecules is likely to involve a multitude of receptor-ligand interactions (Hynes, 1992).

With the above in mind, we studied a panel of adhesion molecules with a view to identifying those receptors which were aberrantly expressed in the colonic neoplastic process. We found that the $\beta 1$ subunit was consistently lost or decreased in the moderately and poorly differentiated carcinomas. In the same tumours the $\alpha 2$ subunit was similarly affected. The $\alpha 2\beta 1$ receptor binds to collagens, laminin and fibronectin depending on the cell type (Kirchhofer *et al.*, 1990). The loss of this integrin in poorly differentiated colorectal carcinomas has been previously reported by one of the authors (Pignatelli *et al.*, 1990*a*), but studies in other tumours and cell lines have reported different findings. Koretz and co-workers (1991) found a marked heterogeneity of expression for this integrin even in normal colonic mucosa with a complete loss of antigen expression in two tumours. No such loss has been seen in pancreatic cancer (Weinel *et al.*, 1992) and a role in tumour invasion has been implicated by Chan *et al.* (1991) in their study on rhabdomyosarcoma cells in which overexpression of $\alpha 2\beta 1$ led to an increased metastatic potential. This may be a reflection on the different ligand specificities of $\alpha 2\beta 1$ already referred to or the differences in epitopes recognised by the monoclonal antibodies used in the various studies. As no correlation to Dukes' stage was found in our study, inferences on the role played by this integrin in invasion and metastasis are difficult to make. The important point to note is that this was a selective integrin loss as the $\alpha 6$ subunit was almost entirely preserved. This latter finding is not in keeping with studies on breast carcinomas nor renal cell carcinomas which show a loss of this subunit in tumours where there is a high degree of loss or disturbance of basement membrane (Pignatelli *et al.*, 1992*b*; Korhonen *et al.*, 1992). The $\alpha 6\beta 1$ integrin is a receptor for the E8 fragment of basement membrane laminin (Lotz *et al.*, 1990), and in view of our apparent contradictory findings for these two subunits, it follows that in colorectal carcinoma $\alpha 6$ complexes with another $\beta$ subunit. There is strong evidence to support the preferential association of $\alpha 6$ with $\beta 4$ in normal and transformed epithelial cells (Sonnenberg & Linders, 1990; Lee *et al.*, 1992) and we suggest that in the colon this may well be the case.

The role of integrins in the loss of differentiation that occurs in most tumours is well-established. Members of the $\beta 1$ integrin subfamily have been implicated in the process of tubule formation in colonic epithelium and recently, functional studies using $\alpha$ specific monoclonal antibodies in colon carcinoma cell lines have identified the $\alpha 2\beta 1$ heterodimer as the key mediator of this process (Pignatelli & Liu, personal communication). Thus, the loss of $\alpha 2\beta 1$ may explain, at least in part, the disturbance of cell polarity and glandular organisation seen in poorly differentiated colorectal adenocarcinomas.

**Figure 11** Stromal changes in expression of $\alpha v$, $\alpha v\beta 3$ & $\beta 5$.

In this paper we report for the first time the distribution of αv, β5 and the heterodimer αvβ3 in colorectal cancer. αvβ3 is the classical vitronectin receptor and elevated expression of this integrin has been associated with invasive melanoma *in vitro* (Felding-Habermann *et al.*, 1992) and *in vivo* (Albelda *et al.*, 1990). Treatment of melanoma cells with an antibody to the integrin causes an increase in their ability to invade basement membrane matrices concomitant with an increase in expression of a matrix degrading enzyme, 72 kDa gelatinase, both at the mRNA and protein level (Seftor *et al.*, 1992). Furthermore, αv appears to be the important subunit responsible for this finding. Our results suggest a stromal distribution for the αvβ3 receptor in colorectal tissue. A moderate proportion (35%) of tumours showed decreased expression of αvβ3 in the stroma, but with preservation of the αv subunit immunoreactivity. Fewer tumours lost this subunit and in four cases there was an increase in expression. The increase in immunoreactivity observed with β5 suggests an altered affinity of the αv subunit. There appears to be a preferential association of αv with the β5 subunit in malignant tissue and a corresponding reduction in expression of the αvβ3 complex. Breast and renal cell carcinomas show a similar association (Pignatelli *et al.*, 1992*b*; Korhonen et *al.*, 1992). It is tempting to speculate that the stromal localisation of the αvβ3/αvβ5 integrins may have a controlling influence over the production and activity of metalloproteinases which would aid in the enzymatic digestion of the extracellular matrix. There is an increasing body of evidence to support the stromal cells as being the source of these enzymes (Poulsom *et al.*, 1992) and thus the nearby localisation of these particular integrins would imply an integral role. This would be in keeping with the results seen in melanoma cell lines (Seftor *et al.*, 1992).

E-cadherin expression was preserved in the well and moderately differentiated carcinomas. However, in all the poorly differentiated carcinomas there was a marked decrease in expression. The same tumours also showed loss of the α2 and β1 subunits. If tumours undergo a progressive loss of differentiation during their genesis and subsequent growth then it would appear from our findings that α2β1 loss is a relatively early event in the dedifferentiation process and E-cadherin loss a later occurrence. Van der Wurff (1992) reported similar findings on E-cadherin in poorly differentiated colorectal carcinomas but also suggested that there was a gradual loss of E-cadherin from adenoma to high grade carcinoma. Our results, although from comparable small numbers, do not support that theory as the decrease in expression of E-cadherin in our series was limited to the least differentiated specimens. Gastric carcinoma shows a similar distribution pattern to that which we found in colorectal carcinoma (Shiozaki *et al.*, 1991). Matsuura *et al.* (1992) have shown that cells from a primary gastric tumour found in ascites show an absence of staining for E-cadherin and decreased intercellular adhesion. This latter finding suggests a role for E-cadherin in the detachment and infiltrative process. Although there was no significant correlation with metastasis in our study the functional activity of the E-cadherin expressed is not known, i.e., immunoreactivity does not imply functional activity. In signet ring cell lines mRNA expression of E-cadherin is preserved even when a down-regulation in protein expression is seen suggesting a defect at the post-translational level. However, it may also be that in some cases a defective protein is produced whose epitopes may still be recognised by the monoclonal antibodies employed but the protein is non-functional. Evidence for this comes from the recent characterisation of a group of proteins termed α-catenins which have been shown to be vital for the intercellular adhesion mediated by E-cadherin (Shimoyama *et al.*, 1992). Cells lacking these proteins but expressing E-cadherin do not adhere to one another, whereas after transfection with the α-catenin cDNA, the same cells acquire strong cell-cell adhesiveness (Hirano *et al.*, 1992). Further evidence for a role of E-cadherin in the invasive process comes from genetic transfection studies. The introduction of a plasmid encoding for E-cadherin-specific antisense RNA into non-invasive transformed cells with high endogenous E-cadherin expression renders them invasive. This implies that the presence of functional E-cadherin may have an invasion suppressor role (Vleminckx *et al.*, 1991).

Carcinoembryonic antigen (CEA) expression was present in normal and malignant tissue. Furthermore, there was accurate basolateral accentuation and some luminal staining in the tumours. CEA is known to be a marker of functional differentiation (Ahnen *et al.*, 1987) and its distribution on the luminal borders is therefore not surprising. The localisation to the basolateral surface implies that this molecule may be interacting with the matrix as well as functioning as an intercellular adhesion molecule. This may be a reflection of the several different forms of CEA that exist, perhaps with different functions (Thompson & Zimmermann, 1988). Its role, therefore, remains speculative, and further elucidation of this is forming the basis of our current studies.

It is clear from the above discussion that many questions remain unanswered about adhesion molecules in the biological behaviour of colorectal carcinoma. From this study we suggest that αvβ3/αvβ5 are largely expressed in the stroma in colorectal tumours and this may be related to the regulation of factors which control the invasive properties of the neoplastic cells, α2β1 integrin loss is a key early event in the neoplastic process and this is related to the dedifferentiation seen in many of these tumours. We also provide further evidence that E-cadherin plays an important role in the differentiation process and that its loss *in vivo* may be a relatively late event when compared to integrin down-regulation.

This work was supported by the Medical Research Council (Project grant: AHGA.1 to M.P., G.W.H.S.) and in part by the Imperial Cancer Research Fund. The authors are grateful to the following for donating the monoclonal antibodies: Sir Walter Bodmer (PR3B10), Dr F. Watt (HAS-6), Dr M. Horton (13C2 and 23C6), Professor M. Takeichi and Dr S. Hirano (HECD-1), Professor J.J. Cassimann (DH12) and Dr M.E. Hemler (β5). We thank Mr P. Clark for statistical advice.

### References

AHNEN, D.J., KINOSHITA, K. NAKANE, P.K. & BROWN, W.R. (1987). Differential expression of carcinoembryonic antigen and secretory component during colonic epithelial cell differentiation and in colonic carcinomas. *Gastroenterology*, **93**, 1330–1338.

ALBELDA, S.M., METTE, S.A., ELDER, D.E., STEWART, R., DAMJANOVICH, L., HERLYN, M. & BUCK, C.A. (1990). Integrin distribution in malignant melanoma: association of the β3 subunit with tumour progression. *Cancer Res.*, **50**, 6757–6764.

BENCHIMOL, S., FUKS, A., JOTHY, S., BEAUCHEMIN, N., SHIROTA, K. & STANNERS, C.P. (1989). Carcinoembryonic antigen, a human tumour marker, functions as an intercellular adhesion molecule. *Cell*, **57**, 327–334.

CHAN, B.M.C., MATSUURA, N., TAKADA, Y., ZETTER, B.R. & HELMER, M.E. (1991). *In vitro* and *in vivo* consequences of VLA-2 expression on rhabdomyosarcoma cells. *Science (Washington DC)*, **251**, 1600–1602.

CROSSIN, K.L. (1991). Cell adhesion molecules in embryogenesis and disease. *Ann. N.Y. Acad. Sci.*, **615**, 172–186.

DAVIES, J., WARWICK, J., TOTTY, N., PHILP, R., HELFRICH, M. & HORTON, M. (1989). The osteoclast functional antigen, implicated in the regulation of bone resorption, is biochemically related to the vitronectin receptor. *J. Cell Biol.*, **109**, 1817–1826.

DE STROOPER, B., SAISON, M. & JASPERS, M. (1988). Monoclonal antibody DH12 reacts with a cell surface and a precursor form of the β subunit of the human fibronectin receptor. *Cell Biol. Int. Rep.*, **12**, 9.

DUBAND, J.-L., DUFOUR, S., HATTA, K., TAKEICHI, M., EDELMAN, G.M. & THIERY, J.P. (1987). Adhesion molecules during somatogenensis in the avian embryo. *J. Cell Biol.*, **104**, 1361–1374.

FEARON, E.R., CHO, K.R., NIGRO, J.M., KERN, S.E., SIMONS, J.W., RUPPERT, J.M., HAMILTON, S.R., PREISINGER, A.C., THOMAS, G., KINZLER, K.W. & VOGELSTEIN, B. (1990). Identification of a chromosome 18q gene that is altered in colorectal cancers. *Science*, **247**, 49–56.

FELDING-HABERMANN, B., MUELLER, B.M., ROMERDAHL, C.A. & CHERESH, D.A. (1992). Involvement of intergrin αv gene expression in human melanoma tumorigenicity. *J. Clin. Invest.*, **89**, 2018–2022.

FIDLER, I.J. & HART, I.R. (1982). Biologic diversity in metastatic neoplasms: origins and implications. *Science (Washington DC)*, **217**, 998–1003.

HEMLER, M.E. (1990). VLA proteins in the integrin family: structure, functions and their role in leukocytes. *Ann. Rev. Immunol.*, **8**, 365–400.

HIRANO, S., KIMOTO, N., SHIMOYAMA, Y., HIROHASHI, S. & TAKEICHI, M. (1992). Identification of a neural α-caterin as a key regulator of cadherin function and multicellular organisation. *Cell*, **70**, 293–301.

HYNES, R.O. (1992). Integrins: versatility, modulation, and signaling in cell adhesion. *Cell*, **69**, 11–25.

HYNES, R.O. & LANDER, A.D. (1992). Contact and adhesive specificities in the associations, migrations and targeting of cells and axons. *Cell*, **68**, 303–322.

KIRCHHOFER, D., LANGUINO, L.R., RUOSLAHTI, E. & PIERSCHBACHER, M.D. (1990). α2β1 integrins from different cell types show different binding specificities. *J. Biol. Chem.*, **265**, 615–618.

KORETZ, K., SCHLAG, P., BOUMSELL, L. & MOLLER, P. (1991). Expression of VLA-α2, VLA-α6 and VLA-β1 chains in normal mucosa and adenomas of the colon, and in colon carcinomas and their liver metastases. *Am. J. Phathol.*, **138**, 741–750.

KORHONEN, M., LAITINEN, L., YLANNE, J., KOUKOULIS, G.K., QUARANTA, V., JUUSELA, H., GOULD, V.E. & VIRTANEN, I. (1992). Integrin distributions in renal cell carcinomas of various grades of malignancy. *Am. J. Pathol.*, **141**, 1161–1171.

LEE, L.C., LOTZ, M.M., STEELE, G.D. & MERCURIO, A.M. (1992). The integrin α6β4 is a laminin receptor. *J. Cell Biol.*, **117**, 671–678.

LOTZ, M.M., KORZELIUS, C.A. & MERCURIO, A.M. (1990). Human colon carcinoma cell use multiple receptors to adhere to laminin: involvement of α6β4 and α2β1 integrins. *Cell Reg.*, **1**, 249–257.

MATSUURA, K., KAWANISHI, J., FUJII, S., IMAMURA, M., HIRANO, S., TAKEICHI, M. & NITSU, Y. (1992). Altered expression of E-cadherin in gastric cancer tissues and carcinomatous fluid. *Br. J. Cancer*, **66**, 1122–1130.

PIGNATELLI, M., SMITH, M.E.F. & BODMER, W.F. (1990*a*). Low expression of collagen receptors in moderate and poorly differentiated colorectal adenocarcinomas. *Br. J. Cancer*, **61**, 636–638.

PIGNATELLI, M., DURBIN, H. & BODMER, W.F. (1990*b*). Carcinoembryonic antigen functions as an accessory adhesion molecule mediating colon epithelial cell-collagen interactions. *Proc. Natl. Acad. Sci. USA*, **87**, 1541–1545.

PIGNATELLI, M., LIU, D., NASIM, M.M., STAMP, G.W.H., HIRANO, S. & TAKEICHI, M. (1992*a*). Morphoregulatory activities of E-cadherin and beta-1 integrins in colorectal tumour cells. *Br. J. Cancer*, **66**, 629–634.

PIGNATELLI, M. CARDILLO, M.R., HANBY, A. & STAMP, G.W.H. (1992*b*). Integrins and their accessory adhesion molecules in mammary carcinomas. *Human Pathol.*, **23**, 1159–1166.

POULSOM, R., PIGNATELLI, M., STETLER-STEVENSON, W.G., LIOTTA, L.A., WRIGHT, P.A., JEFFERY, R.E., LONGCROFT, J.M., ROGERS, L. & STAMP, G.W.H. (1992). Stromal expression of 72 kDa type IV collagenase and TIMP-2 mRNAs in colorectal neoplasia. *Am. J. Pathol.*, **141**, 389–396.

RAMASWAMY, H. & HEMLER, M.E. (1990). Cloning, primary structure and properties of a novel human integrin β subunit. *EMBO J.*, **9**, 1561–1568.

RICHMAN, P.I. & BODMER, W.F. (1987). Monoclonal antibodies to human colorectal epithelium: markers for differentiation and tumour characterisation. *Int. J. Cancer*, **39**, 317–328.

SEFTOR, R.E.B., SEFTOR, E.A., GEHLSEN, K.R., STETLER-STEVENSON, W.G., BROWN, P.D., RUOSLAHTI, E. & HENDRIX, M.J.C. (1992). Role of the αvβ3 integrin in human melanoma cell invasion. *Proc. Natl Acad. Sci. USA.*, **89**, 1557–1561.

SHIMOYAMA, Y., HIROHASHI, S., HIRANO, S., NOGUCHI, M., SHIMOSATO, Y., TAKEICHI, M. & ABE, O. (1989). Cadherin cell-adhesion molecules in human epithelial tissues and carcinomas. *Cancer Res.*, **49**, 2128–2133.

SHIMOYAMA, Y., NAGAFUCHI, A., FUJITA, S., GOTOH, M., TAKEICHI, M., TSUKITA, S. & HIROHASHI, S. (1992). Cadherin dysfunction in a human cancer cell line: possible involvement of loss of α-catenin expression in reduced cell-cell adhesiveness. *Cancer Res.*, **52**, 5770–5774.

SHIOZAKI, H., TAHARA, H., OKA, H., MIYATA, M., KOBAYASHI, K., TAMURA, S. LIHARA, K., DOKI, Y., HIRANO, S., TAKEICHI, M. & MORI, T. (1991). Expression of immunoreactive E-cadherin adhesion molecules in human cancers. *Am. J. Pathol.*, **139**, 17–23.

SONNENBERG, A. & LINDERS, C.J.T. (1990). The α6β1 (VLA-6) and α6β4 protein complexes: tissue distribution and biochemical properties. *J. Cell Sci.*, **96**, 207–217.

STAMP, G.W.H. & PIGNATELLI, M. (1991). Distribution of β1, α1, α2 and α3 integrin chains in basal cell carcinomas. *J. Pathol.*, **163**, 307–313.

TAKEICHI, M. (1991). Cadherin cell adhesion receptors as a morphogenetic regulator. *Science*, **251**, 1451–1455.

TENCHINI, M.L., ADAMS, J.C., GILBERT, C., STEEL, J., HUDSON, D.L., MALCOVATI, M. & WATT, F.M. (1993). Evidence against a major role for integrins in calcium-dependent intercellular adhesion of epidermal keratinocytes. *Cell adhesion and communication*. (in press).

THOMPSON, J. & ZIMMERMANN, W. (1988). The carcinoembryonic antigen gene family: structure, expression and evolution. *Tumor Biol.*, **9**, 63–83.

VAN DER WURFF, A.A.M., KATE, J.T., VAN DER LINDEN, E.P.M., DINJENS, W.N.M., ARENDS, J.W. & BOSMAN, F.T. (1992). L-CAM expression in normal, premalignant and malignant colon mucosa. *J. Pathol.*, **168**, 287–291.

VLEMINCKX, K., VAKAET, L. Jr., MAREEL, M., FIERS, W. & VAN ROY, F. (1991). Genetic manipulation of E-cadherin expression by epithelial tumour cells reveals an invasion suppressor role. *Cell*, **66**, 107–119.

WAYNER, E.A. & CARTER, W.G. (1987). Identification of multiple cell adhesion receptors for type VI collagen and fibronectin in human fibrosarcoma cells possessing unique α and common β subunits. *J. Cell Biol.*, **105**, 1873–1884.

WEINEL, R.J., ROSENDAHL, A., NEUMANN, K., CHALOUPKA, B., ERB, D., ROTHMUND, M. & SANTOSO, S. (1992). Expression and function of VLA−α2, −α3, −α5 and −α6-integrin receptors in pancreatic carcinoma. *Int. J. Cancer*, **52**, 827–833.